# Supplementary material for: EGFR Protein Expression in KRAS Wild-Type Metastatic Colorectal Cancer Is Another Negative Predictive Factor of the Cetuximab Therapy
Source: Cancers (Basel). 2020 Mar 6;12(3):614. doi: 10.3390/cancers12030614 (PMC7139947; doi:10.3390/cancers12030614)
Supplement: Supplementary file 1 [file cancers-12-00614-s001.pdf]

Supplementary Table.1 PFS and OS results by EGFR expression in the primary and metastatic tumors.

	**Primary tumor (N=88)**							
	**No of patients (N)**		**PFS(day)**		P value	**OS(day)**		P value
**HS-Treshold**	**high**	**low**	**high**	**low**		**high**	**low**	
0	83	5	138	231	0.453	418	423	0.492
50	65	23	130	258	0.268	382	512	0.201
100	40	48	98	258	0.131	362	464	0.355
200	6	82	97	147	0.112	67	452	0.042
	Metastasis (N=29)							
	No of patients (N)		**PFS(day)**		P value	**OS(day)**		P value
**HS-Treshold**	**high**	**low**	**high**	**low**		**high**	**low**	
0	26	3	115	430	0.067	354	NR	0.008
50	20	9	119	289	0.024	333	752	0.005
100	13	16	118	203	0.143	333	579	0.018
200	5	24	30	203	0.046	201	518	0.053

NR= not reached
